# The Book World of Medicine and Science

**Published:** 1893-08-12

**Authors:** 


### The Book World of Medicine and Science.

### BOOK REVIEWS.

On Gall Stones and Their Treatment. By A. N. Mayo Robson, F.R.C.S. (London: Cassell and Co.)

A very able and comprehensive manual on a subject not too well understood by the majority of practitioners. Dr. Mayo Robson from his large, and we may even say unequalled, experience in the treatment of this disease is well qualified to speak authoritatively on the subject, and we would highly recommend his work to all medical men, and specially to those engaged in surgical work. The first 70 pages are devoted to anatomical, physiological, and pathological questions, and give a complete *résumé* of all there is known on these subjects. The symptoms, diagnosis, and treatment are gone into in detail, the latter being divided into medical and surgical. Dr. Mayo Robson has never found olive oil to be of the slightest service, although he has tried it in a number of cases. Massage again, although recommended by so high an authority as Dr. Harley, is also condemned, and, we believe, rightly so. Lately Dr. Joseph Sul has stated that he has seen a case where fatal peritonitis was set up by this treatment, and we are inclined to agree with Dr. Robson that we could not recommend it. As he puts it, "I can only say that were I the subject of cholelithrasis, I would not submit to massage, as although it may possibly aid in the expulsion of small calculi, it is impossible to diagnose the absence of large ones, and to know the exact condition of the ducts, which may possibly be ruptured by manipulation." The operation of cholecysotomy, cholelithiotrity, and cholecystectomy are then carefully described, and the arguments for or against each are stated in a clear and judicious manner. The operation of cholecystenterostomy, or the establishment of a fistula between the gall bladder and the intestine is also given. Over 100 pages are then taken up with reports of cases treated by the author, and although agreeing with him that accurate details of individual cases are useful for educational purposes, yet we think that too much space has been taken up by these reports, and that they are given too much in detail. The publishers are also to be congratulated on the general get up of the book, the type used being extremely clear and easily read.

Handbook of Insanity for Practitioners and Students. By Dr. Theodore Kirchhoff, Physician to the Schleswig Insane Asylum, and Private Docent at the University of Kiel. Illustrated with eleven plates. (New York: William Wood and Co.)

Dr. Kirchhoff's book has all the merits that belong to German thoroughness combined with American lucidity. In size it is not too large, nor in style too technical to find a place in the library of the ordinary practitioner, or even of the intelligent layman, philanthropist, or student of humanity, who wishes to comprehend the aberrations which sometimes affect his fellowmen. At the same time its clearness and minuteness of detail, its careful differentiation between allied and often apparently similar forms of mental diseases commend it to the notice of the specialist in insanity. The advice as to treatment is both humane and scientific, such as could only be given by a man whose sympathies as

**THE BOOK WORLD OF MEDICINE AND SCIENCE.**—*(continued).*

well as his intellect were involved in his work. Altogether we regard this as a most compendious, clear, and practical manual of insanity. The plates with which it is illustrated are admirably clear and suggestive, and the general get up of the volume is all that could be desired. One defect only do we note—the work is so admirably rendered in English that the translator's name ought in justice to have been given.

### THE AUGUST REVIEWS.

Fin de Siecle Medicine is the subject of an article in the National Review by Mr. A. Symons Eccles pleading for toleration with experiments "which may not lie strictly within the limits of ancient orthodoxy." The writer comments more fully on the French experiments with a view to supplying defective organs by the injection of materials derived from the same sources in animals, *e.g.*, extract of bone marrow, spinal marrow, sweetbreads, &c. "Perhaps the most remarkable advance in the investigation of the action of organic liquids, as means for the cure or modification of disease, is the alleged discovery by a Russian savant that all the organic liquids derived from different sources, and whose use has been advocated by his French colleagues, depend for their efficiency on a constituent which is common to them all; and it is maintained that all the beneficial effects produced by the injection of diverse organic extracts may be equally derived from a much smaller quantity of a solution containing the active ingredient which is stated may be found in every tissue of the body, but is more easily isolated from some than from others. . . Experience of the remedy in the native country of its inventor has led to its employment in disorders of the nervous system, believed to be dependent on poisonous conditions of the blood, with alleged beneficial results. It is not altogether unsatisfactory to learn that "the use of such suggestions has been regarded with eyes askance by the majority of practitioners of medicine in this country."

Mr. Lafargue La Nouvelle Revue has a very seasonable disquisition on the regeneration of children by the sea, with a summary of the action taken by the authorities in France to furnish seaside hospitals and "convalescent homes" for children. So far as it has gone the accommodation provided cannot be said to be excessive. For the whole of France the number of seaside beds for children is 1,756, and of these over 1,000 are reserved for Parisian children alone. According to M. Lafargue the patients may be counted by hundreds of thousands, and he makes an appeal for the construction of wooden hospitals in favourable localities on the south coast of France for the reception of cases of rickets, scrofula, consumption, &c. Such hospitals he reckons might be constructed at the total cost of under 1,000 francs per bed, and the cost of maintenance he believes might be brought down to the sum of 75 centimes (about sevenpence) per day for each patient. Such an estimate is rather suggestive of a straw a day, and does not give promise of very brilliant results, but M. Lafargue, in his own children's hospital at Banyul, can boast of a proportion of cures of from 80 to 86 per cent., and has evidently practical experience in the cost of living in the localities he names.

